# Perspectives of RAS and RHEB GTPase Signaling Pathways in Regenerating Brain Neurons

**DOI:** 10.3390/ijms19124052

**Published:** 2018-12-14

**Authors:** Hendrik Schöneborn, Fabian Raudzus, Mathieu Coppey, Sebastian Neumann, Rolf Heumann

**Affiliations:** 1 Ruhr-Universität Bochum, Faculty of Chemistry and Biochemistry, Department of Biochemistry II—Molecular Neurobiochemistry, 44801 Bochum, Germany; hendrik.schoeneborn@ruhr-uni-bochum.de (H.S.); fabian.raudzus@ruhr-uni-bochum.de (F.R.); sebastian.neumann@ruhr-uni-bochum.de (S.N.); 2 Laboratoire Physico-Chimie, Institut Curie, CNRS UMR168, PSL Research University, Universite Pierre et Marie Curie-Paris, 75005 Paris, France; mathieu.coppey@curie.fr

**Keywords:** RAS GTPase, RHEB GTPase, Parkinson’s disease, survival, axonal guidance, brain regeneration, optogenetics, magnetogenetics, nanoparticle, Magneto Protein Therapy

## Abstract

Cellular activation of RAS GTPases into the GTP-binding “ON” state is a key switch for regulating brain functions. Molecular protein structural elements of rat sarcoma (RAS) and RAS homolog protein enriched in brain (RHEB) GTPases involved in this switch are discussed including their subcellular membrane localization for triggering specific signaling pathways resulting in regulation of synaptic connectivity, axonal growth, differentiation, migration, cytoskeletal dynamics, neural protection, and apoptosis. A beneficial role of neuronal H-RAS activity is suggested from cellular and animal models of neurodegenerative diseases. Recent experiments on optogenetic regulation offer insights into the spatiotemporal aspects controlling RAS/mitogen activated protein kinase (MAPK) or phosphoinositide-3 kinase (PI3K) pathways. As optogenetic manipulation of cellular signaling in deep brain regions critically requires penetration of light through large distances of absorbing tissue, we discuss magnetic guidance of re-growing axons as a complementary approach. In Parkinson’s disease, dopaminergic neuronal cell bodies degenerate in the substantia nigra. Current human trials of stem cell-derived dopaminergic neurons must take into account the inability of neuronal axons navigating over a large distance from the grafted site into striatal target regions. Grafting dopaminergic precursor neurons directly into the degenerating substantia nigra is discussed as a novel concept aiming to guide axonal growth by activating GTPase signaling through protein-functionalized intracellular magnetic nanoparticles responding to external magnets.

## 1. Introduction

The RAS superfamily is a class of small guanosine triphosphatases (GTPases) comprising more than 150 human members. Based on functional and sequence similarities it is subdivided into the five major subclasses RAS, RHO, RAB, RAN, and ARF [1,2]. In general, RAS signaling is tightly controlled, whereas perturbations in RAS signaling result in malignant tumor formation. In the 1960s, RAS genes were first identified as retroviral oncogenes of Harvey [3] and Kirsten [4] rat sarcoma (H-RAS, K-RAS) viruses. In particular, by identifying constitutively active RAS mutations in human tumors, a new era dawned on RAS research [5]. 

The RAS homolog protein enriched in brain (RHEB) is a member of the RAS subclass that is highly conserved in different organisms from yeast to human [6]. In mammalians, two different *RHEB* genes have yet been identified. The gene products of *RHEB1* (from here on *RHEB*) and *RHEB2* (also *RHEBL1*) share 54% identity and 74% similarity and it is hypothesized that both proteins perform similar functions [7]. RHEB is expressed in various human tissues whereas RHEB2 is mainly expressed in the brain, especially in the cerebral cortex, occipital pole, frontal and temporal lobes [8]. *RHEB* was originally discovered in rat brain as an immediate-early gene, whose cellular level is rapidly increased by high frequency-induced synaptic activity in a *N*-methyl-d-aspartate (NMDA)-dependent manner [9].

In this review, we compare RAS and RHEB GTPases as switch proteins controlling major brain functions such as neuronal survival and regeneration, synaptic connectivity, growth, differentiation, migration, and cytoskeletal integrity. 

After reviewing structural key elements of proteins involved in this switch function and its regulation of downstream intracellular signaling, we will put into focus the importance of subcellular membrane localization culminating in recent aspects of non-invasive optogenetic remote controlling of GTPase signaling after exposing light to brain neurons. Finally, we concentrate on the advances in the fields of manipulating intracellular RAS signaling pathways by generating magnetic guidance cues in neuronal axons containing functionalized intracellular paramagnetic nanoparticles. New therapeutic horizons and possible limits of these novel approaches are critically discussed.

## 2. Structural Basis of the RAS and RHEB GTPase Switch

**Figure 1:**
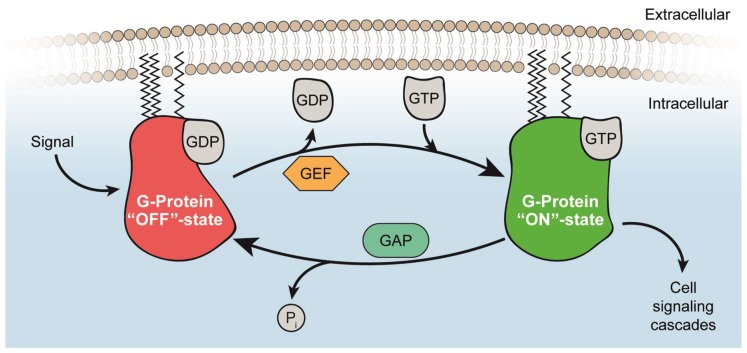
GTPase cycling between GTP-bound “ON” state and GDP-bound “OFF” state. Upon an external signal, a guanine nucleotide exchange factor (GEF) is activating small GTPases (here H-RAS) by exchanging GDP to GTP and thereby modulating cell signaling cascades. To return into the “OFF” state, GTPase-activating proteins (GAPs) accelerate GTP hydrolysis to GDP.

Small GTPases share common biochemical mechanisms and act as conserved binary molecular switches cycling between an active GTP-bound and an inactive GDP-bound state (Figure 1) [10]. Functioning as monomeric or dimeric/oligomeric G proteins [11,12], post-translational modifications of RAS and RHEB specify their subcellular localization resulting in the activation of specific regulators and effectors in a cell type-dependent manner [13,14,15,16]. The proteins of the RAS family of GTPases (H-RAS, N-RAS, and K-RAS) are highly conserved with minor differences in the membrane anchoring hypervariable region (HVR) at the C-terminus [13].

### 2.1. Guanine Nucleotide-Binding

The first 169 amino acids of RAS-related proteins fold into a globular, hydrophilic protein and contain a guanine nucleotide (G)-binding site that consists of a mixed six-stranded β-sheet and five helices executing the central function of nucleotide-binding and hydrolysis [13]. Binding is achieved by the interaction of the nucleotide base with the N/TKXD motif and with the β,γ-phosphates of the conserved phosphate-binding loop (P loop), referred to as GXXXXGKS/T motif. The two nucleotide-bound states are defined by changes in the “switch regions”, first observed in RAS. In the GTP-bound state, two hydrogen bonds from γ-phosphate oxygens link to main chain NH-groups of Thr^35^ and Gly^60^ residues in the switch I and II region of RAS. In particular, Thr^35^ is involved in Mg^2+^-binding whereas Gly^60^ is part of a DXXG motif involved in nucleotide binding. The conformational change is akin to a loaded-spring mechanism due to relaxing switches into the GDP-specific conformation on γ-phosphate release after GTP hydrolysis [10,17].

### 2.2. Activation of RAS and RHEB by GEFs

RAS is activated by guanine nucleotide exchange factors (GEFs) resulting in increased relative levels of GTP-bound RAS [18]. Within a subfamily, GEFs are highly conserved, but in contrast to the guanine nucleotide binding protein (GNBP), structures vary across different families. GEFs for RAS have a cell division cycle 25 (CDC25) domain that forms a trimeric GNBP-nucleotide-GEF state and subsequently results in an intermediary binary complex with RAS having an empty nucleotide-binding site. Thus, the nucleotide-free state is stabilized and preferential rebinding of GTP occurs due to the 10-fold higher concentration of GTP as compared to GDP in the cell.

In detail, GEFs interact with both switch regions and insert residues into the RAS GTPase in close proximity to the P loop and Mg^2+^-binding area to inhibit metal ion- and phosphate-binding. Mg^2+^ is released from its position by Ala^59^ or by GEF amino acid residues. By disturbing residues of the P loop, Lys^16^ is reoriented towards carboxylates of Asp^57^ or Glu^62^ from the switch II region. All together, these conformational changes push the switch II region towards the nucleotide-binding site and switch I outwards of its original position. Thereby, the interaction surface in the phosphate-binding region is perturbed while leaving the base-binding region mostly unperturbed. Consequently, GDP is released, and the nucleotide-free state is stabilized. Subsequently, the entering nucleotide binds and starts to displace the GEF thereby terminating the nucleotide exchange cycle [10,19].

Although there is a high sequence identity between RAS and RHEB, different mechanisms for nucleotide exchange exist. It could be shown that translationally controlled tumor protein (TCTP) is essential for growth and proliferation through regulation of RHEB GTPase in Drosophila [20]. Even though, RHEB may not possess a genuine GEF, as basal nucleotide exchange rates are high enough to sufficiently load RHEB with GTP [21].

### 2.3. Inactivation of RAS and RHEB by GAPs

To switch from the active RAS GTP-bound to the inactive RAS GDP-bound conformation, GTPase-activating proteins (GAPs) accelerate the GTP hydrolysis by several orders of magnitudes so that GDP and P_i_ are released leading to a complete shutdown of signaling within minutes [10,18,22]. Mutations of Gly^12^, Gly^13^, and Glu^61^ near the γ-phosphate in the active site of RAS are crucial for RAS-triggered tumors. Studies performing structural analysis of the RAS-GAP complex in presence of AlF_x_ show that GAP stabilizes the GDP-AlF_x_ state by binding to the catalytic glutamine of the switch II region of RAS. Moreover, for achieving its full activity, GAPs insert an arginine (“arginine finger”) into the active site of the GTPase. Oncogenic mutants of RAS have lost the ability to hydrolyze GTP and thereby remain in a permanently active state [10].

RHEB has been shown to be inactivated by tuberin (TSC2) forming the tuberous sclerosis complex (TSC) together with Hamartin (TSC1). In contrast to RAS-GAP containing an arginine finger, RHEB-GTP-hydrolysis is catalyzed by the presence of an “asparagine thumb” in tuberin [21]. This active-site asparagine corresponds to Asn^1601^ in TSC2, which is mutated in human disease and is also required for TSC2-GAP activity [23].

Taking together, the “ON”/“OFF” state level of RAS is determined by its GEF and GAP activity, while the RHEB “ON”/“OFF” state level is mainly determined by TSC-GAP activities.

### 2.4. Membrane Anchoring of RAS and RHEB

The H-, K- and N-RAS variants differ in the 25 amino acid-comprising HVR containing a tetrapeptide sequence termed as “CAAX (C = Cys, A = aliphatic, and X = any amino acid) motif” at the C-terminus. A part of the post-translational modifications takes place at the CAAX motif leading to trafficking of synthesized RAS from the cytosolic surface of the endoplasmatic reticulum (ER) to the inner surface of the plasma membrane for its anchoring [22]. First, a 15-carbon isoprenyl group is irreversibly attached to RAS^C186^ by farnesyl transferase. At the cytoplasmic ER surface, RAS-converting enzyme (RCE1) and isoprenylcysteine carboxylmethyl transferase (ICMT) mediate the cleavage of AAX tripeptide and carboxylmethylation. For cytoplasmic membrane localization and cellular function of RAS both farnesylation and palmitoylation are required. In addition, H-RAS is palmitoylated on Cys^181^ and Cys^184^ and N-RAS on Cys^181^ in the HVR by palmitoyl transferase at the Golgi apparatus for vesicular transport to the plasma membrane [13].

K-RAS4B is provided with a polybasic region of lysines that interacts with negatively charged lipid head groups of the membrane [24], and even microtubules [25]. Thissen et al. showed the requirement of prenylation and methylation of K-RAS4B for this interaction [26]. Schmick et al. identified the localization of K-RAS to the plasma membrane by solubilization, trapping, and vesicular transport of K-RAS by PDEδ and ARL-2GTP [27]. Ubiquitinated plasma membrane-anchored RAS is unable to activate MAPK pathway and is not targeted to endosomes. [28].

In contrast to RAS which triggers signaling from the inner face of the cytoplasmic membrane, signaling active RHEB is located at endomembrane compartments such as the endoplasmatic reticulum, the Golgi apparatus, and lysosomes [8,29,30,31]. Due to a C-terminal CAAX motif, RHEB is farnesylated at the cysteine residue of the motif by a protein farnesyltransferase which enhances membrane association [29,31].

## 3. Intracellular RAS and RHEB Effector Mechanisms

### 3.1. RAS/RAF Effector Mechanisms

**Figure 2:**
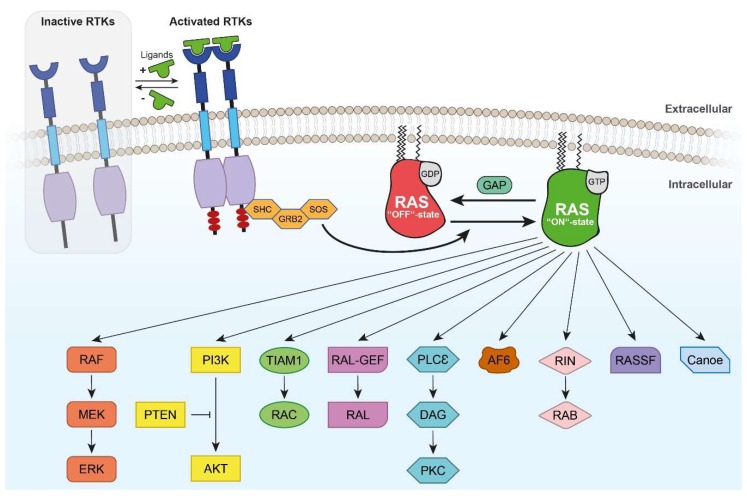
Signaling pathways of RAS GTPase. Receptor tyrosine kinases (RTKs)-mediated activation of RAS in the presence (“+”) and absence (“-“) of ligand is shown as an example. However, multiple upstream regulators of RAS exist. The SHC-GRB2-SOS complex promotes the exchange of GDP to GTP and thereby switching RAS GTPase to the active conformation. Hence, signaling is propagated through the diverse pathways of RAS such as the most prominent RAF/MEK/ERK cascade. RAS-mediated PI3-kinase (PI3K) activation results in protein kinase B (AKT) activation, which is inhibited by phosphatase PTEN (illustrated by T-bar). TIAM1/RAC and RAL-GEF/RAL are involved in cytoskeletal dynamics and cell migration. PLCε/DAG/PKC is associated with Ca^2+^-dependent cellular functions. AF6 forms electrical synapses by localizing at gap junctions. RIN controls axonal pathfinding and via RAB endomembrane signaling. RASSF promotes apoptosis and Canoe regulates cellular polarity.

Effectors for GTP-binding proteins prefer binding to the GTP-bound state involving the switch regions of GNBP which is supported by their structure (Figure 2). The RAS effector rapidly accelerated fibrosarcoma (RAF) binds selectively to the GTP-bound RAS through a ubiquitin-fold domain [10]. RAF is a serine/threonine kinase and is held in an auto-inhibited conformation by the binding of 14-3-3 to phosphorylated Ser^259^ of the RAF amino-terminus [32]. Upon activation by mitogens, GTP-loaded RAS interacts with RAF proteins via RAS-binding domains (RBDs) leading to plasma membrane recruitment of RAF [23]. Autoinhibition is terminated by the interaction of RAS and RAF and correlating with Ser^259^ dephosphorylation and release of inhibitory 14-3-3 [33]. At the membrane, RAF contacts kinases such as SRC family kinases (SFKs) and casein kinase 2 (CK2), which phosphorylate activating sites in the negatively charged N-terminal region. RAS nanoclusters and membrane binding augment the effective RAF concentration thus contributing to RAF dimerization supported by 14-3-3 and its phosphorylated Ser^621^ at the carboxy-terminal tail [34,35]. By dimerization, the catalytic activity of RAF is induced, MEK recruited and phosphorylated, thereby signaling down the three-tiered MAPK modules. A negative feedback loop is implemented by extracellular-signal-regulated kinase (ERK, also referred to as MAPK) signaling through phosphorylation of inhibitory sites in distinct regions of activated RAF, resulting in release from activated RAS and disruption of RAF dimers. RAF-activating and ERK-targeted phosphorylation sites are dephosphorylated to recycle RAF proteins for new activation cycles. Regulation of kinase suppressor of RAS (KSR) occurs in parallel steps. While inactive KSR proteins stay in the cytosol due to the interaction with inhibitory 14-3-3 proteins in their N-terminal region, KSR and MEK form constitutive complexes. Upon dephosphorylation of Ser^406^, 14-3-3 is released and KSR proteins are anchored in the plasma membrane via conserved area 1 (CA1) and CA3. Heterodimerization of KSR and RAF proteins lead to RAF transactivation and MEK-ERK signaling. Phosphorylation of several sites in RAF and KSR by an ERK-mediated negative feedback loop disrupts RAF-KSR dimers and attenuates the signal. Up to now, it is not known whether the N-terminal region of KSR negatively controls the kinase domain or if it is phosphorylated and contributes to activation [32].

Once activated, MEK phosphorylates ERK which dimerizes and translocates to the nucleus activating the E26 transformation-specific (ETS)-family transcription factors by phosphorylation [1].

### 3.2. RHEB Effector Mechanisms

**Figure 3:**
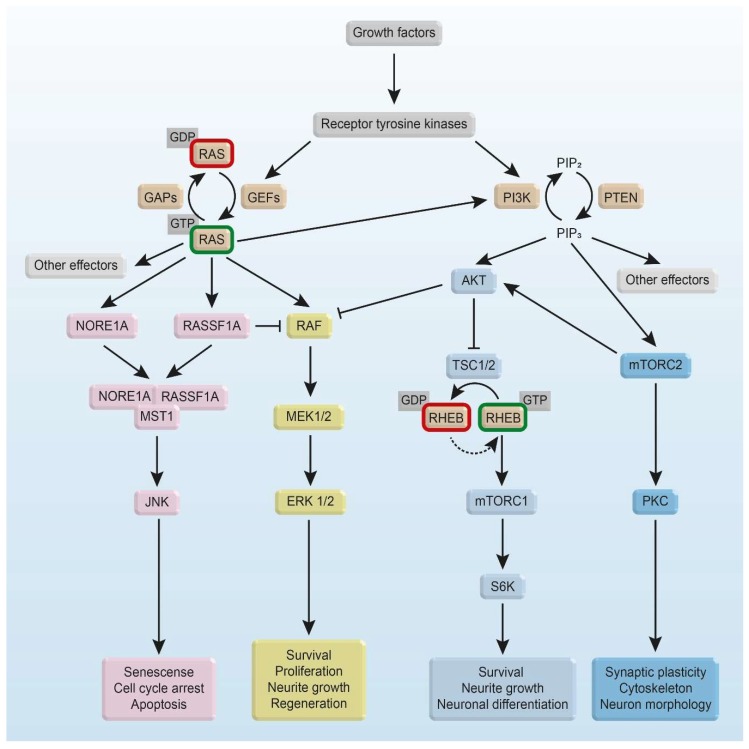
Schematic overview of RAS and PI3K pathway and their interconnection. Binding of growth factors to receptor tyrosine kinases results in activation of RAS and PI3K signaling pathway. Complex-formation of NORE1, RASSF1A, and MST1, together with activated RAS leads to JNK-mediated senescence, cell-cycle arrest or apoptosis [16,84]. RAS-mediated activation of RAF/MEK/ERK signaling plays a role in survival, proliferation, neurite growth, and neuronal regeneration, but the effect differs between various types of neurons [85]. PI3K-mediated phosphorylation of AKT promotes TSC dissociation from lysosomes and RHEB activation. The activation of RHEB by GEF is still under discussion (dashed arrow). Downstream of RHEB, mTORC1 and S6K suppress autophagy and promote survival, neurite growth, and neuronal differentiation [86]. Furthermore, PI3K-mediated mTORC2 activation plays a role in PKC-dependent synaptic plasticity, actin cytoskeleton dynamics, and neuron morphology [87,88]. Strong activation of AKT directly inhibits RAF (illustrated by T-bar) and thereby leading to downregulation of ERK1/2 signaling [89].

The active GTP-bound state of RHEB results in an activation of mammalian target of rapamycin (mTOR) by direct interaction of RHEB with the kinase domain of mTOR (Figure 3) [36]. mTOR is a serine/threonine protein kinase of the phosphoinositide 3-kinase-related kinase family. It is highly evolutionarily conserved and coordinates cell metabolism and growth in response to growth factors, nutrients, and energy levels [37,38]. mTOR interacts with several proteins and forms two distinct complexes: mTORC1 consists of the catalytic mTOR, the mammalian lethal with SEC13 protein 8 (mLST8/GβL), DEP domain-containing mTOR-interacting protein (DEPTOR), the TTI1/TEL2 complex, regulatory-associated protein of mammalian target of rapamycin (RAPTOR), and proline-rich AKT substrate 40 kDa (PRAS40) [30,39,40,41,42,43,44,45,46,47]. mLST8, DEPTOR, and TTI1/TEL2 are also part of mTORC2 along with rapamycin-insensitive companion of mTOR (RICTOR), mammalian stress-activated map kinase-interacting protein 1 (mSIN1), and protein observed with RICTOR 1 and 2 (PROTOR1/2) [39,48,49,50]. mTORC1 directly phosphorylates the eukaryotic translation initiation factor 4E (EIF4E)-binding protein (4E-BP1) and the p70 S6 Kinase (S6K1) [47,51,52,53]. Due to the phosphorylation of these two translational regulators, cap-dependent translation initiation and by this protein synthesis is promoted [54]. mTORC1 further suppresses autophagy by direct phosphorylation of uncoordinated 51-like kinase (ULK1) and autophagy-related gene 13 (ATG13) [55]. mTORC2 is indicated to regulate actin cytoskeleton dynamics by controlling RHO-GEFs [48,56].

### 3.3. Other RAS Effectors

Besides MAPK signaling, RAS is mainly involved in phosphoinositide-3 kinase (PI3K) pathways. The regulatory unit p85, a member of the PI3K class, binds to phosphotyrosine residues of an activated receptor tyrosine kinase. Then, the catalytic subunit p110 is activated and phosphorylates phosphatidylinositol-4,5-bisphosphate (PIP2) to phosphatidylinositol-3,4,5-trisphosphate (PIP3). Alternatively, RAS directly activates PI3K by binding to p110 [57]. PIP3 brings phosphoinositide-dependent kinase1 (PDK1) and AKT serine/threonine kinase (AKT) in close proximity so that PDK1 phosphorylates AKT. Consequently, AKT is involved in cellular processes of growth and prevention of apoptosis. In contrast, phosphatase PTEN stops this signaling by dephosphorylating PIP3 to PIP2 [58].

In addition, the RAS effectors RAL-GEF, T-lymphoma invasion and metastasis-inducing protein 1 (TIAM), RAS association family (RASSF), Canoe, RAS and RAB interactor 1 (RIN1), phospholipase C (PLC), Afadin 6 (AF6), and PKCζ are briefly described to draw a more complete picture of RAS signaling pathways (Figure 2). RAS-like (RAL) family GTPases are ubiquitously expressed in humans and share 80% sequence homology to RAS. RAL guanine nucleotide dissociation stimulator (RALGDS), RAL guanine nucleotide dissociation stimulator-like (RGL), RGL2/RLF, and RGL3 have been shown to activate RAS through binding with RAS association (RA) domains [59]. RAL GTPases regulate cell migration, gene expression, actin organization, and exocytosis and mediate activity-dependent growth of postsynaptic membranes [59,60,61,62,63]. Furthermore, RAL and RHEB GTPase are involved in autophagy, aging, and tumor cell invasion [64]. Moreover, RAL GTPase regulates neurite branching through GAP-43 and activity-dependent growth of postsynaptic membranes [60,65]. The RAC-GEF TIAM1 links the activation of RAS to RAC [66]. Furthermore, TIAM is involved in neurotrophin-3-induced Schwann cell migration [67] and in RAS-related protein 1 (RAP1)-mediated cell spreading [68]. TIAM1 promotes neuronal protrusion and axon guidance through CDC42 and guidance receptor UNC-40/deleted in colorectal cancer (DCC) [69] as well as neurite extension and cell migration [70,71].

RIN1 is a cytosol-localized specific GEF for RAB5/vacuolar protein sorting-associated protein 21 (VPS21) with RA domain and involved in RAS-regulated endocytosis [72]. RIN2 and RIN3 have been detected in endocytic vesicles. In *C. elegans*, RIN1 controls neuronal cell migration and axon pathfinding [73]. PLCε has been identified as a RAS GTPase binding protein which has been discovered by yeast two hybrid screening. PLCε is activated by platelet-derived growth factor (PDGF) and epidermal growth factor (EGF) in a RAS- and RAP1-dependent manner [36,74]. In detail, RAP1 triggers persistent activation of PLCε by PDGF, whereas rapid activation of PLCε is mediated by RAS [74]. Finally, AF6 and PKCζ belong to the family of RAS effectors and are associated with cellular functions such as cell adhesion and transcription [75,76,77,78]. AF6 consists of an actin-binding domain, two RA domains at the N-terminus and a PSD-95 Dlg1 ZO-1 (PDZ) domain allowing interaction with the zonula occludens-1 (ZO-1) tight junction protein [79]. In the rodent brain, the scaffolding protein AF6 together with multi-PDZ domain protein 1 (MUPP1) interacts with connexin36 forming electrical synapses by localizing at gap junctions [80]. Strikingly, AF6 was found in both, maintaining epithelial cell-cell junctions/polarity [75] and negative regulation of RAP1-mediated cell adhesion [76]. For PKCζ, direct interaction with RAS has been shown in vitro and in vivo. The AF6 homolog and RAS-binding protein Canoe is strongly involved in MAPK, int/Wingless (WNT) and Neurogenic locus notch homolog protein (NOTCH) signaling [81]. In Drosophila, the PDZ protein Canoe promotes dorsal closure of the embryo as a RAP1 effector [82] and regulates asymmetric division of neuroblasts [83]. 

## 4. RAS and RHEB Signaling in Survival, Apoptosis, and Neurodegenerative Diseases

### 4.1. RAS Signaling for Survival

Investigating the possible role of RAS signaling in the physiological function of neurotrophic factors, Borasio et al. showed that the intracellular application of RAS protein was sufficient and necessary to promote survival of embryonic peripheral sensory neurons in the absence of nerve growth factor (NGF) [90]. Various mechanisms have been proposed for the RAS-mediated survival activity. In sympathetic neurons, expression of adenoviral constructs coding for constitutively active RAS enhanced neuronal survival by suppressing p53-mediated cell death signaling pathway [91]. In cerebellar granule cells RAS–MAPK signaling appears to promote cell survival by a dual mechanism. It induces expression of pro-survival genes in a cAMP-responsive element-binding protein (CREB)-dependent manner and diminishes cell death by phosphorylating and thereby inhibiting the pro-apoptotic protein Bcl-2-associated death (BAD) [92]. Activity-dependent neuroprotection of cerebellar granule neurons (CGNs) by 100 µM NMDA and 25 mM KCl (K25) is mediated by RAS stimulation through the activation of PI3K-AKT and MEK-ERK pathways [93]. Ca^2+^-induced activation of RAS is regulated by Ca^2+^-sensitive RAS-GEFs and RAS-GAPs as well as RAS effector proteins [94,95].

Insights about the role of enhanced RAS activity in brain neurons were advanced by studies on transgenic mice (named synRas) expressing constitutively active H-RAS^V12^ under the control of a synapsin 1-promoter [96]. Besides hypertrophy of pyramidal neurons in hippocampus and cortex, constitutively active RAS protected motor neurons from degeneration after lesion of the facial nucleus. In addition, survival of tyrosine hydroxylase (TH)-positive neurons of the substantia nigra was enhanced after exposure to dopaminergic neuron-specific toxins [97]. Felderhoff-Mueser et al. showed the resistance of synRas neonates’ brain neurons to toxic hyperoxia treatment [98]. Based on these findings it was concluded, that neuronal RAS signaling induces neuroprotective effects and thereby has a beneficial influence on traumatic brain injury and neurodegenerative disorders such as Parkinson’s disease.

In addition to RAS, PI3K-AKT signaling regulates the development of the neocortex and neuronal survival [99]. PI3K-AKT signaling provides neuroprotective effects by increased expression of anti-apoptotic factors such as B-cell lymphoma-extra-large (BCL-xL) and B-cell lymphoma 2 (BCL-2) through blocking caspase-induced pro-apoptotic pathways [100,101]. Embryonic sympathetic neurons of *Bax* KO mice were transfected with either activated or inhibitory mutants of neurotrophin TRK-receptor effectors to study axon growth in absence of NGF or its downstream signaling mediators. While RAS was necessary and sufficient for NGF-stimulated axon growth, RAF and AKT-induced distinct morphologies. Similar to NGF, active RAF1 caused axon lengthening and AKT lead to increased axon branching and caliber [102]. Furthermore, inactivation of BAD and Forkhead (FKHR) of the pro-apoptotic cascade emphasizes the impact on survival [103].

Using pharmacological inhibitors and dominant-inhibitory mutants of AKT, Dudek et al. showed that AKT is a critical regulator of growth factor-induced neuronal survival [104]. In particular, AKT is involved in both transcription-dependent and -independent control of apoptosis prevention. On the one hand, AKT promotes survival by inhibitory phosphorylation of pro-apoptotic proteins such as BIM, BAX, or BAD and by Forkhead box transcription factor phosphorylation as well as by indirectly inhibiting the tumor suppressor activity of p53 in hippocampal neurons. On the other hand, AKT induces the expression of pro-survival genes by the activation of CREB and nuclear factor κB (NF-κB) [105].

Furthermore, activation of neuronal RAS/MAPK in cultures derived from neurospheres of synRas animals resulted in an activating phosphorylation of CREB and inactivating phosphorylation of BAD. Thus, the transgenic activation of RAS in mouse ventral mesencephalic neurons leads to an increased Nuclear receptor-related 1 protein (NURR1) expression resulting in enhanced dopaminergic properties and a higher number of dopaminergic neurons. In addition, RAS activation counteracted neurotoxic effects of 6-hydroxydopamine (6-OHDA) in dopaminergic neurons [106].

### 4.2. RAS Signaling for Apoptosis

RAS is not only capable of activating cellular transcription, translation, cell-cycle progression, and survival but also apoptosis suggesting that the physiological outcome of RAS activation depends on the cellular context and cell type [5,16]. Cell death plays an important role during the development of the nervous system and in the diseased brain of the adult organism and which is mediated by various signaling pathways, including RAS [107]. Apoptosis, also referred to as programmed cell death, is characterized by nuclear fragmentation, chromatin condensation, and cell shrinkage by an evolutionary highly conserved cellular mechanism [108]. Previously, several publications already demonstrated a link between RAS activity and apoptosis, e.g., RAS inhibition prevents FAS-mediated apoptosis [109]. Furthermore, glioblastoma cells undergo cell death by hyperstimulation of RAS-triggered macropinocytosis [110]. In the rabbit lens epithelial cell line N/N1003A, Ca^2+^-induced RAS activation mediates p53-dependent apoptosis via the RAF/MEK/ERK pathway [111]. Several reports describe the crosstalk between the RAS family and proteins of the BCL-2 family in balancing cell death and cell proliferation [112]. Using neurospheres derived from mice with transgenic activation of RAS (synRas mice), neural cells showed an increased AKT activity resulting in phosphorylation of BAD and thereby inhibiting this pro-apoptotic protein of the BCL-2 family [106].

The RASSF protein family consists of ten members in which RASSF1-8 have multiple splicing variants [113,114,115]. RASSF proteins lack any enzymatic activity and function as scaffolding proteins [116]. Many RASSF proteins contain a N-terminal RA domain for the interaction with RAS [115]. Thereby, the RAS signaling pathway is directly linked to the modulation of apoptosis, autophagy, senescence, inflammation, and DNA repair.

The first identified member of the RASSF family was RASSF5, also known as novel RAS effector 1 (NORE1), is expressed in three isoforms (A–C) resulting from differential promotor usage and alternative splicing. NORE1 selectively binds to RAS in the activated GTP-bounded state (Figure 3) [117] and leads to apoptosis via death receptor-mediated apoptosis [118,119]. The interaction between NORE1 with RAS and mammalian sterile 20-like (MST1/2) kinase is well described. All three isoforms of NORE1 can build a constitutive complex with MST1 in vivo and serum stimulation induces association of NORE1-MST1 with endogenous RAS forming a trimeric complex. In several cell lines, apoptosis is induced by overexpressing constitutively active K-RAS^V12^ or by using the effector loop mutant H-RAS^V12-G37^ resulting in less potent effects, however [120]. Recently, Koturenkiene et al. showed the formation of a stable complex between active H-RAS, NORE1A, and MST1 in vitro without any further factors involved. In addition, they clearly demonstrated that NORE1A has two binding sites: the RBD domain for binding of H-RAS and simultaneously the Salvador-RASSF-Hippo (SARAH) domain for binding of MST1 [121]. Several studies showed that MST kinases are clearly involved in neuronal signaling pathway as well as in the development of diseases in the central nervous system (CNS) [122].

As discussed above, the transgenic expression of activated H-RAS^V12^ in postmitotic neurons promoted neuroprotection after various types of brain lesion and in disease models [96]. In contrast, transgenic expression of H-RAS^V12^ selectively in mature mouse oligodendrocytes resulted in myelin disruption via increased MAPK activity and was associated with nitric oxide production and NOTCH signaling [123]. These different physiological effects in response to enhanced RAS activity (H-RAS^V12^ expression) might be explained by the cell type-specific readouts in neurons or oligodendrocytes, respectively.

Recently, Serdar et al. showed that oligodendrocytes are protected from hyperoxia-induced brain injury due to the transgenic increase of neuronal H-RAS^V12^ in the immature brain of synRas mice. The results suggest that neuronal activation of RAS may generate secondary protective effects in surrounding oligodendrocytes [124]. These studies may have relevance for investigations towards understanding the mechanism of RAS-related genetic diseases. Castello syndrome is a Rasopathy, in which heterozygous activating germ-line mutations in the *H-RAS* gene cause multiple congenital anomalies such as cardiomyopathy, loose skin, coarse face, predisposition to tumors, and mental retardation [125]. In summary, in the brain RAS is involved not only in survival but also in degenerative processes depending on the developmental stage and cell type.

### 4.3. Subcellular Localization-Dependent Signaling of RHEB

RHEB not only influences cellular growth, protein translation, proliferation, and cell cycle but also apoptosis. In absence of e.g., growth factors, the TSC1-TSC2-complex binds to lysosome anchored RHEB-GDP and maintains its GDP-bound state. In presence of growth factors, AKT phosphorylates TSC2, which then dissociates from the TSC1-TSC2-complex and is not able to inhibit RHEB activity anymore [15,126,127]. An additional mechanism of regulating RHEB activation results from amino acid-induced direct binding of the RAS-related small GTPase recombinase-activating gene (RAG) to endomembrane systems [128]. This brings mTORC1 into proximity to RHEB, which allows its activation of mTORC1 by GTP-bound RHEB and leads to further downstream signaling [30]. Conversely, removal of amino acids increases the binding affinity of RAG to TSC1-TSC2-complex and its recruitment to the lysosomal membrane, which facilitates the inactivation of RHEB [129]. Taken together, activation of mTORC1 at the lysosomal membrane needs the synergy of amino acid- and growth factor-mediated signals. Both stimuli play a key role in activation, but neither is sufficient to activate mTORC1 alone [15,86,126,129]. 

Despite the growth factor- and amino acid-mediated activation, mTORC1 is activated by oxidative stress, starvation stress, and ER stress [130,131,132]. The redox-sensitive regulation is found due to an increase of the active GTP-bound state of RHEB by cysteine oxidants. These have only minor impact on mTORC1 localization which leads to the conclusion, that this regulation of mTORC1 is independent of RAG [131]. 

In contrast to oxidative stress, hypoxia-induced stress results in an inhibition of the mTOR pathway. BCL-2/adenovirus E1B 19 kDa interacting protein 3 (BNIP3) is a mitochondrial pro-apoptotic protein member of the BCL-2 superfamily and is activated by hypoxia [133]. Under hypoxic conditions, BNIP3 binds to RHEB resulting in an inhibition of the interaction of RHEB with its downstream effectors as well as a decreased level of RHEB-GTP [134].

### 4.4. RHEB-Mediated Enhancement of Apoptosis

The initial report regarding RHEB showed seizure-induced upregulation of RHEB mRNA in hippocampal neurons [9]. Furthermore, mRNA of RHEB was upregulated upon ultraviolet (UV) irradiation implicating that RHEB could sensitize cells to apoptotic stimuli [135]. This corresponds to the observation that injuries upregulate the expression of RHEB both on mRNA and protein level in the CNS [136,137]. Karassek et al. demonstrated that transient overexpression of RHEB followed by toxic stimuli, i.e., application of tunicamycin, TNFα treatment, or UV irradiation led to enhanced apoptosis in a mTORC1-dependent manner in HeLa cells. Similarly, apoptosis was also enhanced by overexpression of RHEB in primary cortical neurons after excitotoxic stimulation with glutamate. This enhancement was prevented by the knockdown of endogenous RHEB. The pro-apoptotic RHEB signaling was mediated by apoptosis signal-regulating kinase-1 (ASK-1) [138] which is activated by ER stress or oxidative stress [139].

Another mechanism of RHEB/mTOR-mediated enhancement of cellular degeneration results from its negative regulation of autophagy [140]. RHEB activation inhibits autophagy leading to cellular damage, which is especially obvious in neurodegenerative phenotypes when insoluble intracellular aggregates accumulate and induce neuronal death [141,142,143]. Furthermore, permanent activation of RHEB by the cellular loss of TSC1 or TSC2 induces ER stress resulting in unfolded proteins leading finally to apoptosis in tumors and mouse embryonic fibroblasts [144]. Correspondingly, primary hippocampal neurons deficient in TSC2 showed enhanced ER stress-inducing cell death [145]. Moreover, as ER stress is coupled to oxidative stress, the cellular reactive oxygen species (ROS)-levels increased in hippocampal neurons [132]. In general, neurons are more susceptible to oxidative stress than cells of other tissues [146].

It is well described that RHEB activates mTOR kinase, which in turn phosphorylates 4EBP1 and S6K and finally stimulates protein synthesis. Conversely, RHEB can also inhibit protein synthesis by activating protein kinase-like ER kinase (PERK), leading to phosphorylation of the initiation factor eIF2α and thereby inhibiting protein synthesis. This RHEB-PERK pathway is activated under cellular stress, suggesting that cells may respond differently depending on the specific environmental conditions [147].

### 4.5. RHEB in Neurodegenerative Disease

RHEB is involved in neurodegenerative diseases, such as Huntington’s disease (HD). In HD, the protein huntingtin (HTT) contains an expanded poly-glutamine (poly-Q) tract. Typical symptoms of HD are impaired motor and cognitive functions. Under healthy conditions, HTT stimulates signaling through mTORC1. In the case of HD, the poly-Q tract potentiates the signaling because a ternary complex of HTT-RHEB-mTOR is formed, leading to enhanced mTORC1 activity [148]. In contrast to these findings, a reduced striatal mTORC1 activity was observed along with a changed metabolic and degenerative phenotype in the brain of HD patients. Furthermore, constitutively active RHEB increased mitochondrial dysfunction and striatal atrophy influenced the cholesterol homeostasis and impaired dopamine signaling in a mouse model of HD [149].

Another example for the involvement of RHEB in neurodegenerative diseases is Alzheimer disease (AD). A hallmark of AD pathology is the generation of amyloid beta (Aβ) from the amyloid precursor protein (APP) by APP-cleaving enzyme 1 (β-secretase, BACE1) at the β-site of APP. RHEB in its GTP-bound state regulates the stability and activity of BACE1. Protein levels of BACE1 and Aβ generation are suppressed upon RHEB overexpression. The interaction of GTP-activated RHEB with BACE1 stimulates its degradation via the proteasomal and lysosomal pathways. These findings correspond to the pathological situation of AD, as a reduced expression level of RHEB leads to an increased expression of BACE1 in brains of AD patients [150].

More details about the role of RHEB regarding degeneration, regeneration, and connectivity can be found in recent review articles by Ehrkamp et al. and Potheraveedu et al., respectively [16,86]. Taken together, RHEB is not per se involved in apoptosis but only under certain physiological or pathological cellular states.

### 4.6. RAS Signaling and Neurite Growth

During development of the nervous system, neural cells must navigate through a complex and dense packed environment to establish necessary synaptic contacts in the target region. The first event of neuronal morphogenesis is neurite initiation. Neurites are protrusions that emerge from the cell body and are subclassified in axons and dendrites. Each of these cell protrusions forms a growth cone, which extends to filopodia and lamellae. To sense signal molecules and nutrients, these structures are endowed with adequate receptors. To react to these external stimuli, the motility of the growth cone is ensured by strong cytoskeletal dynamics [151,152]. In hippocampal neurons it is shown, that only one axon, but several dendrites can exist at the same time. If this initial axon is transected, in most cases an existing dendrite converts to an axon. Furthermore, dendrites are inhibited to evolve to an axon, if one neuronal process acquired axonal characteristics [153]. However, multiple axon formation is possible by unregulated growth due to GSK-3β inhibition [154,155].

Neurite initiation can be triggered by the binding of neurotrophic factors to receptor tyrosine kinases (RTKs). Experiments with rat pheochromocytoma cells (PC12) show, that the binding of NGF to TRKA receptor is crucial for RAS activation. This further leads to ERK activation, mediated by the protein kinase RAF and MAPK [156,157,158,159]. Clear evidence, that RAS activation is sufficient for neurite outgrowth was provided by experiments in which constitutively active H-RAS^V12^ protein was microinjected in PC12 cells. In these cells, neurite outgrowth was promoted thus showing a neuron-like morphology, which is comparable to the induction with NGF [160]. The intracellular application of H-RAS^V12^ protein in dorsal root ganglion (DRG) neurons showed similar effects [90]. These effects are reversible upon microinjection of anti-H-RAS^V12^ antibodies [160] and do not occur if truncated H-RAS^V12^ is delivered to the cell [90]. 

Deprivation of embryonic peripheral sensory and sympathetic neurons from neurotrophic supply results in cell death thus compromising the investigation on intracellular signaling mechanisms of neurite growth. To enable neurotrophin-independent neuronal survival, experiments were performed with neurons derived from *Bax^−/−^* mice. In these neurons, overexpression of dominant-negative RAS or RAF mutants, as well as pharmacological inhibition of MEK, completely blocks axon growth. Conversely, specific activation of either RAS or RAF is sufficient for sensory axon elongation [102]. Downstream of RAS and RAF signaling, the kinases ERK1 and ERK2 play a key role in neurite growth. Overexpression of the constitutively active fusion protein ERK2-MEK1 is sufficient to phosphorylate ETS domain-containing protein ELK-1 (ELK1) in vivo and to induce ELK1- and activator protein 1 (AP-1)-mediated transcription, resulting in neurite outgrowth in PC12 cells [161]. Despite the translocation of activated ERK2 to the nucleus which leads to the activation of transcription factors, a diffused localization in the cytoplasm and a strong localization at the extending neurites can be observed. This observation suggests that ERK2 phosphorylates distinct effectors at the membrane that contribute to neurite formation [161]. Among these, ERK substrates such as the gap junction protein connexin [162,163], cytoskeletal proteins such as microtubule-associated proteins (MAPs) [164,165], TAU [166], and vinexin [167] are discussed.

In addition to RAF, the PI3K-AKT-pathway is activated by RAS but with different effects on neurite morphology [102]. PI3K is shown to be required for axon growth in sympathetic neurons [168]. In sensory neurons, selective activation of PI3K or AKT does not significantly contribute to axon elongation but leads to a doubled diameter of E13 axons [102]. PI3K activation is localized to the growth cone where it inactivates GSK-3β. In addition, PI3K downstream target integrin-linked kinase (ILK) supports the inactivation of GSK-3β directly by phosphorylation [169]. Due to the inactivation of GSK3-β, its downstream target adenomatous-polyposis-coli (APC) is not phosphorylated and can bind to the plus- ends of microtubules in the growth cone which increases microtubule stability [170]. Furthermore, inactivated GSK-3β cannot phosphorylate the collapsin response mediator protein-2 (CRMP-2), which allows axon formation by mediating microtubule assembly [171] and modulating microtubule dynamics [172]. PI3K also activates RAC1 at the plasma membrane which leads to the activation of the WASP-family verprolin homologous protein (WAVE) complex and further to actin polymerization mediated by the ARP2/3 complex [173]. 

Although there is some knowledge about the mechanisms of neurite growth, in the adult CNS spontaneous regrowth is very limited, if neurons lose their axons due to injury or disease. In contrast to this, neonatal CNS axons are capable to regenerate after being injured [174,175]. These observations led to the suggestion to use peripheral nerves as a support to overcome the inhibitory cues preventing long-distance regeneration in the CNS [176,177]. In addition, these experiments raised the question, whether the reactivation of developmental growth mechanisms could empower adult CNS axons to regenerate again [178]. It has been shown that the application of growth factors could rescue neuronal death after axotomy [179,180] which brought again into focus the RAS-ERK and PI3K-AKT pathways [181]. Further pathways that play a role in regeneration will not be discussed in detail but have been excellently elaborated in recent reviews [182,183].

### 4.7. TRK Receptor-Mediated RAS/RAF/ERK Pathway

Animal models overexpressing tropomyosin receptor kinase B (TRKB) in adult layer V motor cortex neurons showed, that upon subcortical aspiration lesions, these neurons successfully regenerated, and their axon grew towards brain-derived neurotrophic factor (BDNF)-secreting subcortical grafts. In contrast, overexpression with a truncated TRKB mutant which is not capable to activate the ERK/MEK pathway did not lead to neuronal regeneration [184]. Expression of fibroblast growth factor 2 (FGF-2) in retinal ganglion cells significantly leads to regenerative axon growth after a microcrush lesion of the optic nerve. This effect is also dependent on ERK activation and can substantially be blocked by administration of a specific ERK inhibitor (PD980590) [185]. Despite its anti-apoptotic and neuroprotective effect [186], overexpression of BCL-2-associated athanogene-1 (BAG1) promotes axon regeneration of retinal ganglion cell (RGC) after optic nerve crush. It is shown, that BAG1-overexpression activates RAF-1 and induces translocation from the membrane to the cytoplasm followed by RHO-associated protein kinase 2 (ROCK2) translocation from the membrane to the perinuclear region. However, overexpression of MEK1 is not sufficient for axon regeneration after optic nerve injury, although ERK1/2 is phosphorylated [187].

Recent studies underlined the importance of B-RAF in axon growth and regeneration. Overexpression of conditional active B-RAF in mice was sufficient to rescue nociceptor axon extension in the absence of TRKA signaling. After dorsal root crush injury, overexpression of conditional active B-RAF in DRGs resulted in axons that penetrate the dorsal root entry zone (DREZ) and grew into the spinal cord, whereas wild type DRGs stopped at the DREZ as they respond to its growth-inhibitory signals. Similar results were obtained for RGC axons after optic nerve crush. When RAF downstream effector kinases MEK1 and MEK2 are knocked out, regenerative axon growth induced by constitutive active B-RAF is substantially inhibited [188]. By silencing suppressors of mTOR pathway such as PTEN, TSC1, and TSC2, axon regeneration after injury is likewise stimulated in CNS and peripheral nervous system (PNS) neurons [189,190,191,192]. Furthermore, regeneration is successfully blocked by administration of mTOR inhibitor rapamycin, which implicates an important role of the mTOR pathway [189]. The combination of overexpression of B-RAF and silencing of PTEN leads to enhanced regenerative axon growth by a synergistic effect of B-RAF and PI3K signaling [188].

### 4.8. Other Intracellular Mechanisms Signaling for Regeneration

Besides B-RAF and PI3K signaling, other genetic and epigenetic changes in injured neurons are discussed to play an important role in regeneration. RHO GTPases such as Ras homolog gene family, member A (RHOA), CDC42, and RAC1 play a crucial role in neurite growth by regulation of the actin polymerization [193]. RHOA inhibits neurite growth in response to neurite outgrowth inhibitor (NOGO), myelin-associated glycoprotein (MAG), whereas administration of the RHOA inhibitor C3 improved neurite outgrowth. Furthermore, inhibition of RHOA downstream effector ROCK showed comparable results. In contrast, CDC42 and RAC1 are positive regulators of axon growth and induce lamellipodia and filopodia formation [193,194]. Damage of the axonal membrane results in an increased influx of calcium ions [195] and higher levels of the second messenger cAMP [196]. The increased cAMP levels then activate pro-regenerative proteins such as CREB1 [197] and dual leucine zipper-bearing kinase (DLK) [198]. Poly(ADP-ribose) polymerase 1 (PARP1) is activated when neurons are exposed to growth-inhibiting molecules such as MAG, myelin protein NOGO and myelin membrane-associated chondroitin sulfate proteoglycans (CSPGs) [199]. Activation of PARP1 leads to increased poly(ADP-ribose) levels, resulting in limited axonal growth [200], but pharmacological inhibition or genetic deletion of PARP1 is not sufficient to promote axonal growth alone [201]. In addition, axonal regeneration can be enhanced by knocking out the ribonucleic acid (RNA) processing enzyme 3′-terminal phosphate cyclases A and B (RTCA and RTCB), which are important for RNA repair and stress-induced mRNA splicing [202,203]. Analysis of transcriptional changes in different neuronal cell lines revealed that transcription factors such as p53, c-Jun, ATF3, CREB, STAT3, NFATs, NF-κB, SOX11, SNON, and the Krüppel-like family of transcription factors (KLF) family are involved in axon growth and axon regeneration. In particular, NF-κB has been shown to be involved in regrowth of the genetically damaged hippocampus [204]. These regenerative activities are highly dependent on the cellular or genetic context. Upregulation of transcription factors that are promoting axon growth only occurs in early development or in cells that are capable of regeneration. Furthermore, anti-axon growth factors dominate when both, growth enhancing and growth suppressing transcription factors are upregulated [205]. Epigenetic changes, such as histone or DNA modifications in the context of developmental axon growth is highly investigated, but the role in regeneration is not clearly understood, yet [182].

As axon growth is controlled by an orchestra of enhancing and suppressing stimuli, more recent studies try to target a combination of different pathways. mTOR activation by expression of constitutively active RHEB was shown to enhance neuronal survival and axon growth of substantia nigra dopaminergic neurons after a neurotoxin-induced lesion [206]. After spinal cord injury, the expression of constitutively active RHEB is not sufficient for axonal regeneration. In combination with CSPG digestion, the number of propriospinal axons that are capable of reinnervating distal spinal cord significantly increased, and sprouting was enhanced [207,208].

## 5. Optogenetics

Optogenetics combines genetic and optical methods to control biological processes in living systems, ranging from single cells over tissues to organisms [209]. With optogenetic tools, the vision to understand cellular functions, dynamics and decision-making processes extended considerably. In addition, intensity and timing of signals depending on optogenetic assays allow dissecting molecular networks and quantitative understanding of information flow as well as its functional mapping in a native cellular context.

### 5.1. Light-Directed Protein-Protein Interaction Devices

Photosensitive switches are proteins that have been modified to regulate a wide variety of signaling pathways in living cells through conformation changes in response to light. Mainly, the plant proteins cryptochrome [210,211], light-oxygen-voltage (LOV) [212] and phytochromes [213,214], as well as the fluorescent protein DRONPA [215], have been applied as reversible optogenetic systems to control signaling pathways [216]. Cryptochrome 2 (CRY2) is a protein isolated from *Arabidopsis thaliana* which uses a ubiquitously expressed endogenous flavin as a chromophore. Upon exposure to blue light (405–488 nm), CRY2 homooligomerizes [217] and binds to its interaction partner cryptochrome-interacting basic helix-loop-helix 1 (CIB1) within seconds. CRY2 resets in the dark to its initial state within 5 min after activation [211]. The LOV domains from different organisms are all sensitive to blue light (440–473 nm) using flavin as their chromophore. In the dark, the flavin-binding domain binds to its carboxy-terminal helical extension (Jα). Light exposure results in a covalent binding of the flavin chromophore and Cys^450^ leading to the release of its autoinhibitory conformation [218]. Alternatively, the LOV domain fused to the DNA-binding domain of Gal4 homodimerizes and binds to DNA upon blue light activation to regulate gene expression [219]. In addition, the LOV2 domain has been used to cage a peptide epitope and thereby blocking the binding of its interaction partner PDZ. This opens the possibility to regulate a vast number of PDZ-dependent cellular functions [220]. Furthermore, Guntas et al. embedded the SSRA peptide in the C-terminal helix of the LOV2 domain from *Avena sativa* to create a system being highly efficient in changing binding affinity upon light stimulation. This avoids cross-reactions with other molecules in the cell and is therefore used in a variety of organisms for protein recruitment. In the dark, LOV2 domain prevents the SSRA peptide from binding to its partner SSPB, while upon blue light stimulation the C-terminal helix undocks and thus allows SSPB binding. By computational protein design, phage display, and high-throughput binding assays, the affinity for SSPB has been increased by over 50-fold to generate an improved light-inducible dimer (iLID) [221,222]. 

For gaining deeper insights, CRY2/CIB1, iLID/SSPB, and LOVpep/PDZb were compared. In vivo colocalization and functional assays showed dramatic differences in the dark and lit state binding affinities correlating with changes in transcription control and GTPase signaling [223].

Phytochrome B (PHYB) is a protein that is activated by red light at 650 nm and inactivated by infrared light at 750 nm within seconds [224]. In cells expressing chromophore-free apo-PHYB protein, a light-sensitive system is generated as soon as phycocyanobilin (PCB) is bound. This chromophore is present in photosynthetic organisms, while in non-photosynthetic organisms, PCB must be synthesized by recombinant enzymes or directly delivered to cells [225,226]. Upon red light illumination, the PHYB-PCB complex alters conformation and binds to the phytochromes interacting factor (PIF) thus activating the desired signaling pathway [213].

Another mechanism is described by using DRONPA which is a photoactivatable fluorescence protein whose quaternary structure is changed by light activation independent of small-molecule chromophores. Upon photoactivation at 390 nm, DRONPA switches from a monomeric “dark state” to a dimer in the “fluorescent state” thereby inhibiting the function of fused protein of interest. Illuminating with light at a wavelength of 490 nm, the conformational change can be reversed and DRONPA is converted back to a monomer. Thus, observing DRONPA fluorescence enables to track the extent of protein inhibition [215,216].

### 5.2. Light-Mediated Activation of RAS/RAF/ERK

As discussed above MAPK/ERK-mediated signaling plays a key role in cell differentiation, proliferation, apoptosis as well as survival [227]. To activate MAPK-signaling pathways by light has been first shown in yeast by recruiting the scaffold protein STE5 to the membrane and thereby activating MAPK. Upon blue light stimulation, MAPK signaling was activated by binding of STE5 fused to a PDZ domain to a membrane-anchored LOV-epitope fusion protein [220]. Meanwhile, the iLID system has been combined with the two RHO GTPase family members RAC and CDC42 to induce lamellipodia and directed migration on a fibronectin substrate [228]. 

Based on a PHYB-PIF6 system, MAPK-signaling cascade was induced by light in mammalian cells. The catalytic domain of RAS-GEF SOS (SOScat) was fused to PIF6 whereas PHYB was anchored to the membrane. Upon red light-induced PHYB-PIF6 binding and SOScat recruitment to the membrane, the RAS/MAPK (ERK) pathway was activated by transient (20 min) or sustained (120 min) illumination. Response was measured by a high-throughput proteomic method monitoring total- and phospho-protein levels. A group of proteins was defined that were phosphorylated or upregulated after both transient and sustained RAS activation such as members of the PLC/PKC signaling pathway and P90RSK. In addition, other proteins exclusively reacted to the sustained RAS activation such as members of the mTOR pathway, STAT3, and zinc finger protein SNAI1 (SNAIL). Taken together, these methods combine distinct temporal patterns of RAS activation with proteome profiling [229]. 

RAF/MEK/ERK light-mediated activation has also been shown by using the CIB1-CRY2 system. CRY2 was fused to RAF1 and CIB1 anchored to the plasma membrane. Upon blue light stimulation, RAF1 membrane recruitment leads to an activation of RAF1 and its downstream targets. In the absence of NGF, RAF1 signaling activation induced neurite outgrowth in PC12 cells. Astonishingly, permanent ERK activation was not required, while periodic on/off light stimulation was sufficient to support maximum neurite outgrowth, showing a 45 min unrevised threshold for the light-off phase [230]. Using the CRY2-C-RAF/CIB1 system as light-switchable ERK activation system, Aoki et al. showed the propagation of ERK activity pulses to the neighboring cells [231] and induction of collective cell migration [232]. In a different study, the heterodimerization of CRY2-RAF1 and CIB1-RAF1 blocked RAF-RAF interaction by steric effects thus preventing ERK activation [233]. Recently, Dine et al. engineered FGFR1-OptoDroplets to convert transient local inputs to persistent local clustering and cytoskeletal contraction, thus harnessing spatial memory in RTK signaling [234].

Goglia et al. demonstrated the control of RAS activity by “Opto-SOS”, using the PHY-PIF system. Functional control of “Opto-SOS” expression was monitored with a live-cell reporter imaging ERK responses. This revealed a highly quantitative input-to-output map of the signaling pathway [235]. “Opto-RAF”, is a light-mediated tool to activate RAF and to mimic RAS-triggered dimerization, which allows studying the effect on RAF signaling induced by B-RAF and C-RAF hetero- and homodimer formation. The B-RAF and C-RAF protein kinase inhibitors vemurafenib and dabrafenib suppress oncogenic B-RAF^E600^ kinase activity. Opto-RAF activity was enhanced by dabrafenib at a low dose and inhibited at a high dose. Thus, vemurafenib has been identified as a paradoxical activator of B-RAF and C-RAF homo- and heterodimers [236]. In PC12 cells, RAF kinase induced cell differentiation by light-controlled RAF/MEK/ERK signaling using the CRY2 protein together with the N-terminal domain of cryptochrome-interacting basic-helix-loop-helix (CIBN). Applied to *Xenopus* embryos, RAF kinase could be reversibly activated at any desired developmental stage in specific cell lineages dependent on light stimulation [237].

Taking together, the activation of the RAS/RAF/MAPK pathway using light-switchable protein-protein interaction devices is a versatile tool to control cell differentiation, migration, and neurite outgrowth in a spatiotemporal manner.

### 5.3. Light-Mediated Activation of PI3-Kinase

Regulation of PI3K is involved in cellular functions such as survival, growth, migration, and progression [238]. Phosphorylation of PIP3 by PI3K has been coupled with light-inducible systems thereby activating downstream pathways such as protein kinase C (PKC), RAC, and AKT. Activation of the PI3K-PIP3 signaling pathway by light has been shown by the PIF6-PHYB membrane recruitment system [239] as well as the CIB1-CRY2 protein aggregation [240]. In addition, the CIB1-CRY2 protein system has been used to regulate the phosphoinositide metabolism, leading to local activation of the 5-Ptase phosphatase and by this to a loss of membrane ruffling, whereas recruiting PI3-kinase to the membrane produced PIP3 [241].

### 5.4. Combined Activation of RAS- and PI3K-Pathways

Kim et al. controlled the activity of the fibroblast growth factor receptor (FGFR) and its three downstream signaling pathways RAS/RAF/ERK, PI3-kinase, and PLC by illumination with blue light on a second-based time-scale through CRY2 oligomerization. CRY2 was inserted between the cytosolic catalytic domain of FGFR and a membrane-targeting sequence. Upon light stimulation, CRY2 oligomerization caused autoactivation of the receptor and subsequently activation of all three downstream signaling pathways [242]. The chimeric receptor Opto-mFGFR1 was generated by fusing the LOV domain of Aureochrome 1 from *Vaucheria frigida* to the intracellular catalytic domain of murine FGFR1 and thus showing MAPK/ERK pathway activation upon blue light-stimulated dimerization of LOV domains [243]. 

Ono et al. studied the photoactivated expression of differentiation-associated markers in the channelrhodopsin-2 expressing OS3 (OS3ChR2) cells that are clonal bipotential glial progenitor cells. Channelrhodopsin-2 is a blue light-sensitive cation channel for the control of ion influx. Upon blue light activation, increased Na^+^- and Ca^2+^-ion concentrations led to ERK1/2 signaling which resulted in cell growth and survival [244]. Dependent on the light intensity, Opto-CNK1 stimulated RAF/MEK/ERK or AKT signaling in Michigan Cancer Foundation 7 (MCF7, human breast adenocarcinoma cell line) cells. At low light intensity Opto-CNK1 induced differentiation by stimulating ERK activity, whereas triggering AKT signaling stimulated cell proliferation at higher light intensity [245].

### 5.5. Activating the RHO Family of Proteins

Optogenetics has also been applied to other related protein classes such as RHO GTPases including its members RAC1, CDC42, and RHOA to modify and regulate cellular functions such as cell motility, organelle development, and actin dynamics [227]. RAC1 has been fused to the LOV domain of phototropin and activated by unwinding the helix linking LOV as a photo-cage system. Using 458 nm or 473 nm light, RAC1 could be repeatedly and reversibly photoactivated and generated localized cell protrusions and ruffling. By this, cell motility and the direction of movement was controlled [218]. Moreover, RAC1 was fused to flavin-binding, kelch repeat, f box 1 (FKF1) that contains a LOV domain and a light-detecting flavin mononucleotide (FMN). Blue light-induced activation of FKF1 to plant specific nuclear protein GIGANTEA (GI) resulted in translocation to the membrane and increased lamellipodia formation [246]. Levskaya et al. spatiotemporally activated RHO GTPases by light-controlled membrane recruitment of GEFs, e.g., Intersectin (ITSN), TIAM, and TIM. GEFs were linked to PIF and PHYB anchored in the plasma membrane. Light-induced membrane recruitment and protein interaction using various optogenetic systems promoted activation of Intersectin and TIAM leading to the generation of lamellipodia in NIH3T3 cells. Recruiting TIAM Dbl-homology domain (DH)/pleckstrin-homology domain (PH) domains to the plasma membrane using different photoactivatable switches led to protrusions of varying size. In particular, in NIH3T3 cells TIAM allowed local induction and protrusion of lamellipodia [223,224]. Zhou et al. linked the CDC42-GEF Intersectin to the DRONPA system enabling membrane recruitment as well as photo-caging of cellular signaling mediators and enzymes. Introducing a Lys^145^ to Asn^145^ mutation into DRONPA, the homotetrameric complex monomerized upon blue light illumination, which was reversed by violet light. Tandem fusions of the monomeric DRONPAK form and of the tetrameric mutant DRONPAN allowed the reversible dissociation of intramolecular dimers upon blue light stimulation. This photo-uncaging of ITSN caused lengthening of pre-existing filopodia [215,247].

Due to the restricted light penetration into deep brain regions, patient-directed brain regeneration therapies using optogenetic tools are limited. Interestingly, in a recent study by Chen et al., optogenetics has been combined with nanoparticles (UCNPs) absorbing and upconverting the tissue-penetrating near-infrared (NIR) light into visible light to enable remote-controlled optical neuron therapy in the mouse brain. UCNPs stimulate deep brain neurons upon transcranial NIR light illumination evoking dopamine release from genetically modified neurons in the tegmental area [248].

## 6. Magnetogenetics

Remote control of cellular functions in a spatiotemporal manner constitutes an important challenge in fundamental and biomedical research. Recently, physical and chemical techniques have been combined to address genetic and pharmacological issues leading to new approaches in cell behavior control and, consequently, to regenerative medicine applications. Several studies demonstrated the control of cell-lineage differentiation [249], gene expression [250,251], intracellular organelle transport, and positioning [252] as well as directed migration and motility [218,253,254]. New efforts emerged by developing approaches to remotely control biological functions based on magnetic stimulation [255].

Mainly, three research areas arose to investigate the effect of magnetic fields on cellular response. First, there is the research on organisms that can navigate by detecting magnetic fields, referred to as “magnetosensation” [256,257]. Second, investigating the influence of strong magnetic fields on biological systems and their processes have been studied to show the response of biopolymers to external magnetic stimulation. Finally, besides their conventional use as drug-delivery systems [258,259,260,261,262] or imaging contrast agents [263,264], functionalized magnetic nanoparticles (MNPs) have been applied to remote-control cellular function and signaling pathways as well as biological events. In particular, this technique combines beneficial effects of deep and non-invasively penetration of magnetic fields into tissues, organisms, and cultured cells with precise modulation of cellular events. We focus in this part on recent progress and applications of magnetic nanoparticles as a promising approach in fundamental and biomedical research, termed as “magnetogenetics” [255].

### 6.1. Functionalized Magnetic Nanoparticles in Regenerative Medicine

Approaches using MNPs to activate or transmit cellular signaling by stretching, twisting, or bending the plasma membrane gained significant influence in recent years. Application of MNPs was expanded to manipulate biological processes and cellular behavior towards tissue engineering and regenerative medicine [265]. MNPs allow precise control with millisecond and submicrometric resolution in single protein assays [266]. Seo et al. used mono-functionalized magnetic zinc-doped ferrite nanoparticles with dielectric silica layer and plasmonic gold shell to force a response of NOTCH receptor and VE-cadherin signaling at the surface of human bone osteosarcoma epithelial (U2OS) cells [250]. Furthermore, magneto-mechanical stimulation with MNPs allows direct gating of ion channels by stretching or deflection. With cube-shaped MNPs bound to stereocilia bundles, Lee et al. achieved ultrafast mechanical control of deflection-relaxation dynamics in inner ear hair cells [267]. By fusing genetically encoded ferritin nanoparticles to the transient receptor potential cation channel subfamily V member 4 (TRPV4), action potentials in neurons were triggered by Ca^2+^-transients upon applying a static magnetic gradient. Moreover, reward behavior in mice and tactile behavior in zebrafish were affected [268]. Anti-Frizzled functionalized MNPs were used to investigate mechano-stimulation of the WNT pathway activation in human mesenchymal stem cells (hMSC). Remote control of anti-Frizzled-MNPs by an oscillating magnetic bioreactor shows an activation of the WNT/β-catenin pathway by a TCF/LEF luciferase reporter assay and demonstrating nuclear localization of β-catenin [269].

Tseng et al. established a technique for massively parallel mechanical modulation of single-cell behavior by growing magnetic nanoparticle-loaded HeLa cells on defined micro-magnetic patterns. After magnetic manipulation and accumulation of nanoparticles, a highly coordinated response was observed leading to p21-activated kinase (PAK)-dependent filopodia stimulation and oriented cell division [270].

### 6.2. Magneto-Thermal Approaches

Besides magneto-mechanical stimulation, MNPs are used for magneto-thermal approaches to convert field stimulation into heat by placing suitable MNPs in a radio-frequency magnetic field [271]. The commonly used transient receptor potential (TRP) plasma membrane ion channels respond to cold (8–28 °C, i.e., TRPA1 and TRPM8) [272,273] or higher (41–43 °C, i.e., TRPV1, TRPV3, and TRPV4) temperatures [274]. Magneto-thermal stimulus allowed control of Ca^2+^-influx by TRPV1 gating in HEK293 cells or lead to triggering of action potentials in hippocampal neuronal cells [275,276]. Huang et al. first used heating of MNPs in *C. elegans* near sensory neurons to cause thermal avoidance. Gating of the TRPV1 channel was achieved by fusing them to iron oxide MNPs using biotin-streptavidin interactions and targeting a high concentration of the complexes to the cellular membrane which reduced side effects and increased the heating efficiency of ion channels [275]. Chen et al. showed neuronal excitation in mice by activation of TRPV1 in ventral tegmental brain regions through high concentrations of freely diffusing MNPs in the cytoplasm [276]. Ferritin and synthetic magnetic nanoparticle enable further control of insulin expression and, consequently, blood glucose level in mice [251,277]. Controlling neuronal activity by the ferritin-TRPV1 system in hypothalamic glucose-sensing neurons in mice allowed regulating insulin levels and metabolism [278].

### 6.3. Magnetic Control of Receptor Clustering

Finally, MNPs can control cellular functions and biological processes in the cell based on their concentration and spatial distribution. Initially, MNPs bound to membrane receptors triggered their signaling pathways by inducing oligomerization in the plasma membrane upon the magnetic stimulus. Mannix et al. bound MNPs coated with monovalent ligands to transmembrane receptors and accumulated them in the plane of the membrane by bead-bead attraction. By this, clustered FcεRI-dinitrophenyl receptor-ligand complexes initiated local inflammatory response and increase of cytosolic calcium [279]. Inducing dimerization of EGF-receptor using biotin-streptavidin coupled EGF-antibodies to MNPs activated the corresponding downstream pathway [280]. By binding iron oxide MNPs to the death receptor 4 (DR4), a magnetic switch was generated, which promotes apoptosis signaling upon magnetic stimulation in vitro. Moreover, the magnetic switch allowed the remote control on a micrometer-scale leading to morphological changes of a zebrafish tail [281].

### 6.4. Magnetic Activation of Intracellular Signaling

Even more challenging is the intracellular control of cellular processes and signaling due to nanoparticle delivery into the cytoplasm without endosomal recycling and surface passivation to guarantee colloidal stability inside the cytoplasmic environment. The functionalization of MNPs with proteins advancing to intracellular nanoactuators that activate signaling pathways by interaction with their effectors allow manipulating complex signaling cascades on a subcellular level. In response to an external magnetic field, the nanoactuators can be localized and accumulated at any desired location in the cell [282]. Particularly, biofunctionalized MNPs induced and maintained protein gradients inside living cells with high spatial and temporal resolution [283]. Moreover, superparamagnetic iron oxide nanoparticles (SPIONs) can affect signal transduction pathways depending on their surface coatings. Small negatively charged SPIONs mimic physiological growth factors through ERK and AKT activation by promoting proliferation of RAS-transformed breast epithelial cells with the same efficiency as EGF signaling [284].

Accumulating the RAC1-GEF TIAM coupled to 500 nm MNPs at the plasma membrane by a magnetic tip lead to cell protrusion and actin cytoskeleton remodeling [285], whereas concentration-dependent RAN-GTP-coupled MNPs initiated faster growth in droplets of *Xenopus* eggs extract by RAN/RCC1 signaling [286].

## 7. Outlook and Perspectives: The Dawn of Magneto Protein Therapy in Brain?

Axonal regeneration in the adult diseased mammalian brain is required to bridge the interrupted connectivity between the neuronal cell body and the distally innervated target region. Endogenous guidance mechanisms permitting axonal regrowth over large distances are usually not sufficient for functional recovery. Activation of RAS and RHEB GTPase signaling pathways have been shown to support regeneration in various model systems using intracellular applied proteins or transgenic expression in cells or animal models. Global neuronal activation of RAS GTPases protects neurons from degeneration and promote sprouting even into growth-inhibitory regions of the brain [86,289]. However, to recover the correct and functional brain connectivity, a non-invasive method is lacking for achieving directed axonal growth into the target region over long distances.

Optogenetic approaches allowing focal activation of fiber growth by light led to new mechanistic insights in cultured neuronal cells and in animals. Although penetration of light into the brain is limited, upconverting nanoparticles that absorb tissue-penetrating NIR light and emit wavelength-specific visible light were recently used to stimulate dopamine release of genetically tagged neurons even in deep brain regions [248].

Promising magnetogenetic approaches have been achieved to mimic activation of receptors such as WNT, NOTCH, and EGF demonstrating their potential in regenerative medicine. Unfortunately, the above-mentioned advances do not allow to resolve the basic requirement for remote control of directed fiber growth for circuit recovery in the diseased brain.

In neurodegenerative diseases, lost neurons can be replaced by transplantation. Experiments show that human induced pluripotent stem cell (iPSC)-derived dopaminergic progenitor cells survived and functioned as midbrain dopaminergic neurons in a primate model of Parkinson’s disease (*Macaca fascicularis*) [290]. Histological studies demonstrated that the dopaminergic neurons transplanted into the striatal target regions extended dense neurites. Accordingly, there was an increase in spontaneous movement after toxic treatment with 1-Methyl-4-phenyl-1,2,3,6-tetrahydropyridin (MPTP). Based on these advancements, human trials using stem cell-derived dopamine neurons have been initiated, recently [291].

**Figure 4:**
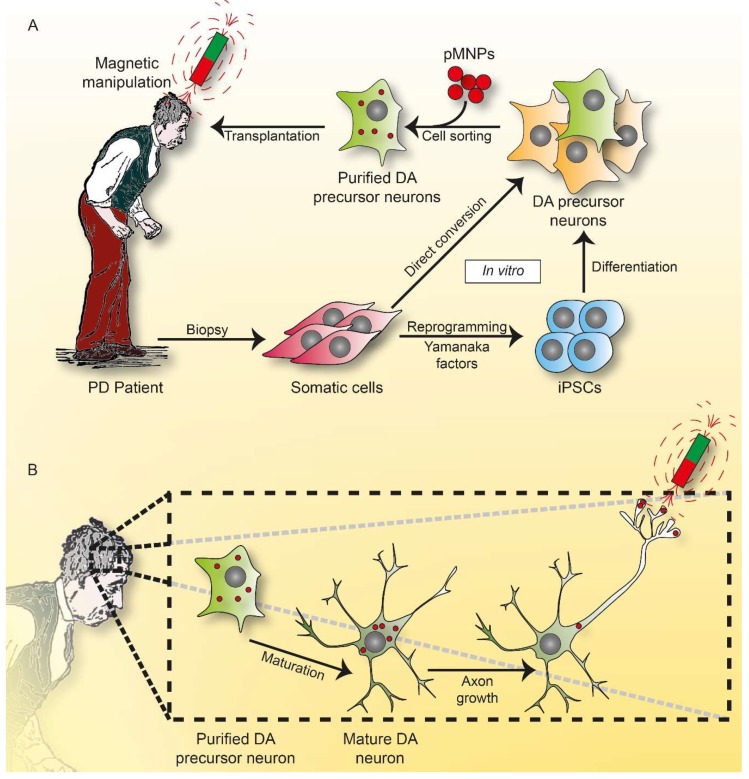
Concept of Magneto Protein Therapy. (**A**) Somatic cells obtained from Parkinson’s patient by biopsy are reprogrammed [287] or directly converted [288] to dopaminergic (DA) precursor neurons in vitro. After cell sorting and loading with protein-functionalized magnetic nanoparticles (pMNPs), these cells are grafted to the striatal target regions or substantia nigra, where neurons degenerate. (**B**) In the patient’s brain, the grafted DA precursor cells convert into mature DA neurons. By the application of an external magnetic field, pMNPs are driven towards the inner membrane of the growing axon thereby activating small GTPases allowing spatially controlled fiber growth.

Encouraged by these achievements, future efforts should be made to transplant dopamine precursors directly into the substantia nigra where the neuronal loss occurs in Parkinson’s disease. As a prerequisite, guided axonal growth must be achieved in transplanted neuronal axons from the substantia nigra to distally located and denervated striatal target regions. A novel approach is envisaged by loading precursor neurons with protein-functionalized magnetic nanoparticles before transplantation. These nanoparticles are thought to activate suitable GTPases from inside the neuronal membrane and guide them towards the desired target by applying external magnets. This concept is currently part of the EU-project MAGNEURON (Horizon 2020, FET Open, grant agreement No. 686841; Figure 4).

In contrast to most magnetogenetic experiments described in this review, this above-mentioned concept avoids genetic manipulation in patients. Protein therapy is currently emerging in clinical applications, e.g., by using cell penetrating peptides [292]. Even in cellular models of Parkinson’s disease, intracellular protein delivery has been shown to protect toxin-induced neuronal cell death [293]. Here, we propose the application of protein-functionalized MNPs as a novel tool to control intracellular signaling for neuronal regeneration. We call this approach “Magneto Protein Therapy”, abbreviated MPT. Ideally, Magneto Protein Therapy, magnetogenetic and optogenetic tools will help to resolve the major problem towards enhancing functional recovery of the lost brain connectivity in neurodegenerative disease.

## Abbreviations

4E-BP1	Eukaryotic translation initiation factor 4E (EIF4E)-binding protein
6-OHDA	6-hydroxydopamine
AD	Alzheimer disease
AF6	Afadin 6
AKT	Protein kinase B
AP-1	Activator protein 1
APC	Adenomatous-polyposis-coli
APP	Amyloid precursor protein
ARF	ADP ribosylation factor
ASK-1	Apoptosis signal-regulating kinase-1
ATG13	Autophagy-related gene 13
Aβ	Amyloid beta
BAD	Bcl-2-associated death
BAG1	BCL-2-associated athanogene-1
BAX	Bcl-2-associated X protein
BCL-2	B-cell lymphoma 2
BCL-xL	B-cell lymphoma-extra large
BDNF	Brain-derived neurotrophic factor
BIM	Bcl-2-like protein 11
BNIP3	BCL-2/adenovirus E1B 19 kDa interacting protein 3
CA	Conserved area
CAAX	C = Cys, A = aliphatic and X = any amino acid
CDC	Cell division cycle
CDC25	Cell division cycle 25
CGN	Cerebellar granule neuron
CIB1	Cryptochrome-interacting basic helix-loop-helix 1
CIBN	Cryptochrome-interacting basic-helix-loop-helix, N-terminal domain
CK2	Casein kinase 2
CNK1	Connector enhancer of kinase suppressor of RAS 1
CNS	Central nervous system
CREB	cAMP response element-binding protein
CRMP-2	Collapsin response mediator protein-2
CRY2	Cryptochrome 2
CSPG	Chondroitin sulfate proteoglycan
DCC	Deleted in colorectal cancer
DEPTOR	DEP domain-containing mTOR-interacting protein
DH	Dbl-homology domain
DLK	Dual leucine zipper-bearing kinase
DR4	Death receptor 4
DREZ	Dorsal root entry zone
DRG	Dorsal root ganglion
EGF	Epidermal growth factor
EIF4E	Eukaryotic translation initiation factor 4E
ER	Endoplasmatic reticulum
ERK	Extracellular-signal-regulated-kinase
ETS	E26 transformation-specific
ETS	E26 transformation-specific
FGF	Fibroblast growth factor
FGFR	Fibroblast growth factor receptor
FKF1	Flavin-binding, kelch repeat, f box 1
FKHR	Forkhead
FMN	Flavin mononucleotide
GAP	GTPase-activating protein
GDP	Guanosine diphosphate
GEF	Guanine nucleotide exchange factor
GNBP	Guanine nucleotide binding protein
GTP	Guanosine triphosphate
GTPases	Guanosine triphosphatases
HD	Huntington’s disease
hMSC	Human mesenchymal stem cells
H-RAS	Harvey-RAS
HTT	Huntingtin
HVR	Hypervariable region
ICMT	Isoprenylcysteine carboxylmethyl transferase
iLID	Improved light-inducible dimer
ILK	Integrin-linked kinase
ITSN	Intersectin
KLF	Krüppel-like family of transcription factors
KO	Knock out
K-RAS	Kirsten-RAS
KSR	Kinase suppressor of RAS
LOV	Light-oxygen-voltage
MAG	Myelin-associated glycoprotein
MAPK	Mitogen activated protein kinase
MEK	MAPK/ERK kinase
mLST8	Mammalian lethal with SEC13 protein 8
MNPs	Magnetic nanoparticle
MPT	Magneto Protein Therapy
MPTP	1-Methyl-4-phenyl-1,2,3,6-tetrahydropyridin
mSIN1	Mammalian stress-activated map kinase-interacting protein 1
MST	Mammalian sterile
mTOR	Mammalian target of rapamycin
MUPP1	Multi-PDZ domain protein 1
NF-κB	Nuclear factor κB
NGF	Nerve growth factor
NIR	Near-infrared
NMDA	*N*-Methyl-d-aspartic acid
NOGO	Neurite outgrowth inhibitor
NORE1	Novel RAS effector 1
NOTCH	Neurogenic locus notch homolog protein
N-RAS	Neuroblastoma-RAS
NURR1	Nuclear receptor-related 1 protein
PAK	p21-activated kinase
PARP1	Poly(ADP-ribose) polymerase 1
PC12	Pheochromocytoma cells
PCB	Phycocyanobilin
PDK1	Phosphoinositide-dependent kinase1
PDZ	PSD-95 Dlg1 ZO-1
PERK	Protein kinase-like ER kinase
PH	Pleckstrin-homology domain
PHYB	Phytochrome B
PI3K	Phosphoinositide-3 kinase
PIF	Phytochromes interacting factor
PIP2	Phosphatidylinositol-4,5-bisphosphate
PIP3	Phosphatidylinositol-3,4,5-trisphosphate
PKC	Protein kinase C
PLC	Phospholipase C
PNS	Peripheral nervous system
PRAS40	Proline-rich AKT substrate 40 kDa
PTEN	Phosphatase and tensin homolog
RA	RAS association
RAB	RAS-like proteins in brain
RAC	RAS-related C3 botulinum toxin substrate
RAF	Rapidly accelerated fibrosarcoma
RAG	Recombination-activating gene
RAL	RAS-like
RALGDS	RAL guanine nucleotide dissociation stimulator
RAN	RAS-like nuclear
RAP1	RAS-related protein 1
RAPTOR	Regulatory-associated protein of mammalian target of rapamycin
RAS	Rat sarcoma
RASSF	RAS association family
RBD	RAS-binding domain
RCE1	RAS-converting enzyme
RGC	Retinal ganglion cell
RGL	RAL guanine nucleotide dissociation stimulator-like
RHEB	RAS homolog protein enriched in Brain
RHO	RAS homologs
RHOA	Ras homolog gene family, member A
RICTOR	Rapamycin-insensitive companion of mTOR
RIN	RAS and RAB interactor
RNA	Ribonucleic acid
ROCK	RHO-associated protein kinase
ROS	Reactive oxygen species
RTK	Receptor tyrosine kinases
S6K1	p70 S6 Kinase
SARAH	Salvador-RASSF-Hippo
SFK	SRC family kinase
SNAIL	Zinc finger protein SNAI1
SPIONs	Superparamagnetic iron oxide nanoparticles
TCTP	Translationally controlled tumor protein
TH	Tyrosine hydroxylase
TIAM1	T-lymphoma invasion and metastasis-inducing protein 1
TNFα	Tumor necrosis factor α
TRK	Tyrosine receptor kinase
TRKB	Tropomyosin receptor kinase B
TRP	Transient receptor potential
TRPV4	Transient receptor potential cation channel subfamily V member 4
TSC	Tuberous sclerosis complex
TSC1	Hamartin
TSC2	Tuberin
U2OS	Human bone osteosarcoma epithelial cell line
ULK1	Uncoordinated 51-like kinase
VPS21	Vacuolar protein sorting-associated protein 21
WAVE	WASP-family verprolin homologous protein
WNT	int/Wingless
ZO-1	Zonula occludens-1
